# Measuring gender when you don’t have a gender measure: constructing a gender index using survey data

**DOI:** 10.1186/s12939-016-0370-4

**Published:** 2016-05-28

**Authors:** Peter M. Smith, Mieke Koehoorn

**Affiliations:** Institute for Work & Health, 481 University Avenue, Suite 800, Toronto, ON M5G 2E9 Canada; School of Public Health and Preventive Medicine, Monash University, Melbourne, Australia; Dalla Lana School of Public Health, University of Toronto, Toronto, ON Canada; School of Population and Public Health, Faculty of Medicine, University of British Columbia, 2206 East Mall, Vancouver, BC V6T 1Z3 Canada

**Keywords:** Gender, Sex, Labour force, Gender roles, Measurement, Survey data

## Abstract

**Background:**

Disentangling the impacts of sex and gender in understanding male and female differences is increasingly recognised as an important aspect for advancing research and addressing knowledge gaps in the field of work-health. However, achieving this goal in secondary data analyses where direct measures of gender have not been collected is challenging. This study outlines the development of a gender index, focused on gender roles and institutionalised gender, using secondary survey data from the Canadian Labour Force survey. Using this index we then examined the distribution of gender index scores among men and women, and changes in gender roles among male and female labour force participants between 1997 and 2014.

**Methods:**

We created our Labour Force Gender Index (LFGI) using information in four areas: responsibility for caring for children; occupation segregation; hours of work; and level of education. LFGI scores ranged from 0 to 10, with higher scores indicating more feminine gender roles. We examined correlations between each component in our measure and our total LFGI score. Using multivariable linear regression we examined change in LFGI score for male and female labour force participants between 1997 and 2014.

**Results:**

Although women had higher LFGI scores, indicating greater feminine gender roles, men and women were represented across the range of LFGI scores in both 1997 and 2014. Correlations indicated no redundancy between measures used to calculate LFGI scores. Between 1997 and 2014 LFGI scores increased marginally for men and decreased marginally for women. However, LFGI scores among women were still more than 1.5 points higher on average than for men in 2014.

**Conclusions:**

We have described and applied a method to create a measure of gender roles using survey data, where no direct measure of gender (masculinity/femininity) was available. This measure showed good variation among both men and women, and was responsive to change over time. The article concludes by outlining an approach to use this measure to examine the relative contribution of gender and sex on differences in health status (or other outcomes) between men and women.

## Background

Better understanding and accounting for male and female differences has been gaining attention in many health-related research areas [1–3]. In the area of work and health, men and women differ in their work exposures and work-related health conditions. In addition, the relationships between work exposures and health outcomes may also differ for men and women. These male/female differences can be due to sex – referring to biological differences between men and women – or gender – referring to social differences between men and women [4].

It is increasingly recognised that both sex and gender matter in understanding the relationships between working conditions and health outcomes, and that research that fails to take sex and gender into account is limited in both quality and applicability [4, 5]. Stratifying analyses to examine the relationships between work and health separately for men and women has been proposed as one approach to better account for sex and gender [6]. However, it is recognised that this approach does a better job of understanding “sex” differences than it does in understanding “gender” differences [7, 8]. Furthermore, sex and gender often interact, suggesting that differences between men and women might be due to a combination of both biological (sex) and social/cultural (gender) factors. To develop a better understanding of the relative contribution of each of these aspects requires measures of both sex and gender to be included in analyses [5, 8].

Measuring gender and sex can take different forms depending on the way data are being collected. When conducting primary data collection for quantitative studies researchers have the options to include measures of gender, such as the Bem-Sex-Role-Inventory (BSRI) [9] which asks participants to self-identify with personal traits, or the Masculine Gender Role Stress scale [10]. However, are there options for researchers to measure gender if such scales are not present in existing data?

The concept of gender diagnosticity was first introduced by Lippa and Connelly [11] to estimate the probability of being male or female, based on some gender-related diagnostic indicator. In their original study, Lippa and Connelly used occupational preference ratings as a measure of gender-diagnosticity, finding that these preferences were distinct from responses to the Personal Attributes Questionnaire (PAQ) [12] and the BSRI [9]. They also reported that occupational preference was more predictive of being a man or a woman than either the BRSI or PAQ indices [11]. This suggests that a gender diagnostic approach offers an alternative method to measure social differences between men and women based on their roles and preferences, compared to indices such as the PAQ and BRSI that are based on gender stereotypes [11].

A decade later Lippa and colleagues used this same approach (occupational preferences) to examine the role of sex and gender on mortality [13]. In this study they found masculinity (as measured by occupational preferences) was predictive of mortality among both men and women, resulting in the highest mortality rate being observed among the most masculine men, and the lowest mortality rate observed among the most feminine women [13]. The objective in a gender-diagnostic approach is to identify indicators that best differentiate between different gender-based groups. As Lippa and Connelly noted in their original paper, multiple indicators would ideally be used to form a gender index, resulting in a more reliable scale [11].

Most recently a variation on this approach has been used to examine the impact of sex and gender on cardiovascular risk factors among individuals with premature acute coronary syndrome [14]. In this study the gender index was comprised of information on whether the respondent was the primary earner in their household; their personal income; the number of hours and responsibility for housework; and level of stress at home – along with measures of masculinity and femininity from the BSRI [14]. Similar to the previous gender diagnostic studies, this paper found that both sex and gender were important in predicting many cardiovascular risk factors, but that the gender score was generally more important than sex (male/female) in predicting risk in multivariable models [14].

The preceding studies relied on primary data collection where the concept of a gender index or gender-diagnostic approach was part of the study design. This is not always feasible in population-health or health services research studies that rely on existing surveys and administrative health records, despite the growing body of literature that indicated that gender differences matter to primary prevention and health care practices [15–17].

In this paper the aim is to develop a gender-index using the Canadian Labour Force Survey (LFS). The LFS was selected because it has a number of questions that are commonly available in other data sources, and a gender-index using this data may be readily applied (and modified or expanded upon) to other secondary data sources. We use data from the 1997 and 2014 Labour Force Surveys to accomplish three objectives: to develop a gender-index using existing population health survey data; to examine the distribution of our gender index across males and females (i.e. to ensure that it measured a separate concept to sex); and to examine if there have been changes in gender roles (as measured by the index) among male and female labour market participants between 1997 and 2014. We then discuss how this index (or a similarly constructed index) could be used in research that exploits secondary data to better understand the relative contribution of aspects of gender and sex in male/female differences in health outcomes.

## Methods

### Data source

This paper uses secondary data from Statistics Canada’s LFS. The LFS is a monthly survey carried out by Statistics Canada with the objective of providing information on trends in labour market participation and hours of work across major occupational and industrial sectors in Canada [18]. The LFS surveys approximately 56,000 Canadian households per month. Households remain in the sample for six consecutive months, with one sixth of the sample rotated out, and replaced by a new group of households representing one sixth of the sample each month. The target population for the LFS is the civilian, non-institutionalised population 15 years of age and over residing in all of Canada’s provinces and territories. Persons living on Aboriginal reserves, full-time members of the Canadian Armed Forces, and the institutionalised population are excluded from coverage, as are households in extremely remote areas. Statistics Canada estimates these groups represent less than 2 % of the Canadian population aged 15 and over, and that the LFS is representative of its target population [18]. For the purpose of this analysis, the Public Use files from the 1997 and 2014 Labour Force Surveys were used through Statistics Canada’s Data Liberation Initiative [19]. The year 1997 was chosen as the start point for the analysis, as the questions asked in the LFS changed in this survey year. For each survey cycle, the analysis was restricted to respondents who were currently working for pay or profit in the past month, excluding unpaid family workers, regardless of the number of hours worked.

### Labour Force Gender Index (LFGI)

Gender is a multidimensional construct that includes four dimensions: gender roles (behavioural norms applied to men and women); gender identity (how an individual sees themselves on the male/female continuum); gender relationships (how individuals are treated by others based on their ascribed gender); and institutionalized gender (how power and influence are distributed differently among men and women) [4]. The LFGI constructed from the LFS focused primarily on the dimensions of gender roles and institutionalised gender among labour force participants. Given the data available, the LFGI was comprised of four main measures: responsibility for caring for children; occupational segregation; hours of work relative to partner/spouse; and education relative to partner/spouse. Differences in male and female participation rates in education in Canada and other developed countries have changed considerably since the early 1970’s,with women outnumbering men in university and post-secondary education completions [20, 21]. However, education was used in the construction of the LFGI as it is a measure of educational attainment relative to one’s partner/spouse, not a measure of absolute educational attainment. Each measure in the index is described in detail below.

#### Responsibility for caring for children

In each cycle of the LFS respondents are asked if they were away from work (either completely or partially) in the last week, and the reason for this absence, with one option being personal or family responsibilities. Respondents working less than 30 h per week are also asked the main reason they are not working more hours per week, with one option being caring for children and another being other personal or family responsibilities. Using responses to these questions, the following three category variable was created: 0 = no reduction in labour market participation due to personal or family responsibilities; 1 = part or full week absence due to personal or family responsibilities; 2 = working part-time due to personal or family responsibilities.

#### Occupational segregation

Self-reported occupation is coded into 47 major groups based on the National Occupational Classification system [22]. For the LFGI responses to the 1997 LFS were used to classify each of these 47 occupations into one of four groups: 0 = occupations where less than 26 % of workers were women; 1 = occupations where 26 to 50 % of workers were women; 2 = occupations where 51 to 74 % of workers were women; and 3 = occupations where 75 % or more of workers were women. Occupations with the lowest participation of women are conceived as the most masculine occupations, while occupations with the highest participation of women are conceived as the most feminine occupations.

#### Hours of work relative to partner/spouse

Respondents are asked the usual number of hours they usually work each week. For respondents who are living with a spouse they are also asked the number of hours their spouse usually works per week. Using both these sources of hours worked each respondent was grouped into one of the following four categories: 0 = respondent working, but spouse not in the labour force; 1 = respondent working more hours than their spouse; 2 = respondent working the same number of hours as their spouse; and 3 = respondent working less hours than their spouse. If respondents did not have a spouse they were grouped with respondents working more hours than their spouse.

#### Education level relative to partner/spouse

Respondent’s and spouse’s highest level of education are reported in the following six categories: 0 to 8 years of education; some secondary education; graduated from high school; some post-secondary education; post-secondary certificate or diploma; and university degree. Using this information respondents were grouped into one of the following three categories: 0 = respondents with a higher level of education than their spouse; 1 = respondents with the same level of education as their spouse; 2 = respondents with a lower level of education than their spouse. Similar to work hours, respondents without a spouse were grouped with respondents with a higher level of education than their spouse.

To create the LFGI the values for the above four measures (caring for children, occupational segregation, hours or work and education level) were summed for each respondent providing a score ranging from 0 to 10, with higher scores indicating more traditionally feminine gender labour market roles of respondents and lower scores indicating more traditionally masculine gender labour market roles.

### Analysis

Correlations between the four measures of the LFGI were examined, and between each component and the final LFGI score. LFGI scores were then compared for men and women, and for the 1997 and 2014 LFS. Linear regression analyses then examined if the relationship between sex (male versus female) and LFGI scores changed between 1997 and 2014, after adjustment for differences in age, province, and month of survey participation between the 1997 and 2014 surveys. To examine if gender scores had changed for men and women between 1997 and 2014 a multiplicative interaction term between sex (male/female) and survey year was included in the model. The regression analysis was based on a 10 % random sample to avoid the possibility of a Type I error given the size of the LFS samples. All analyses were weighted to account for the initial probability of selection for each household, non-response and coverage errors, as specified by Statistics Canada [18]. Analyses were conducted using SAS Version 9.3 [23].

## Results

Table 1 presents the distribution of each of the LFGI measures for men and women in the 1997 and 2014 Labour Force Surveys. Women were more likely to have taken time off and be working part time due to household responsibilities in both 1997 and 2014, and they were also more likely to be working fewer hours than their spouse in both time periods. As expected, given that occupation categories were based on 1997 labour market participation, we observed women were more likely to be working in occupations with a greater proportion of women, and men in occupations with a greater proportion of men. Distribution across occupational segregation groups for men and women only changed to a small extent between 1997 and 2014. Differences in education were also noted for men and women, although these were smaller in magnitude than observed for other measures. In 1997 an almost identical proportion of men and women had lower levels of education than their spouse (conceptualised as being the most feminine category), but by 2014 men were more likely than women to have lower education than their partner/spouse.

**Table 1:** Distribution of gender index components for Canadian men and women in 1997 and 2014

	1997 LFS (*N* = 696,350)			2014 LFS (*N* = 729,132)		
	Men	Women	*p*-value for diff	Men	Women	*p*-value for diff
Responsibility for caring for children						
No absence from work due to family or household responsibilities	98.8 %	91.1 %	< 0.001	98.0 %	91.3 %	< 0.001
Part or full-week absence due to family or household responsibilities	1.0 %	2.7 %		1.7 %	4.5 %	
Works part-time due to family or household responsibilities	0.2 %	6.3 %		0.3 %	4.2 %	
Occupation (based on 1997 LFS only)						
Less than 26 % women	45.3 %	7.5 %	< 0.001	46.0 %	7.7 %	< 0.001
26 to 50 % women	29.8 %	22.7 %		27.7 %	21.8 %	
51 % to 74 % women	22.0 %	43.4 %		22.4 %	45.5 %	
75 % women	2.9 %	26.5 %		3.9 %	25.0 %	
Hours of work						
Respondent works spouse does not	18.9 %	7.9 %	< 0.001	15.1 %	8.6 %	< 0.001
Respondent works more than spouse/respondent does not have a spouse	62.6 %	41.8 %		63.4 %	46.3 %	
Respondent works same amount as spouse	13.5 %	16.2 %		15.0 %	15.5 %	
Respondent works less than spouse	5.0 %	34.2 %		6.6 %	28.6 %	
Education						
Respondent higher level of education than spouse/respondent does not have a spouse	20.2 %	18.9 %	< 0.001	14.5 %	18.6 %	< 0.001
Respondent same education as spouse	62.2 %	63.3 %		68.1 %	69.0 %	
Respondent lower education than spouse	17.6 %	17.9 %		17.5 %	12.4 %	

Respondents to Statistics Canada’s Labour Force Survey

**Fig. 1:**
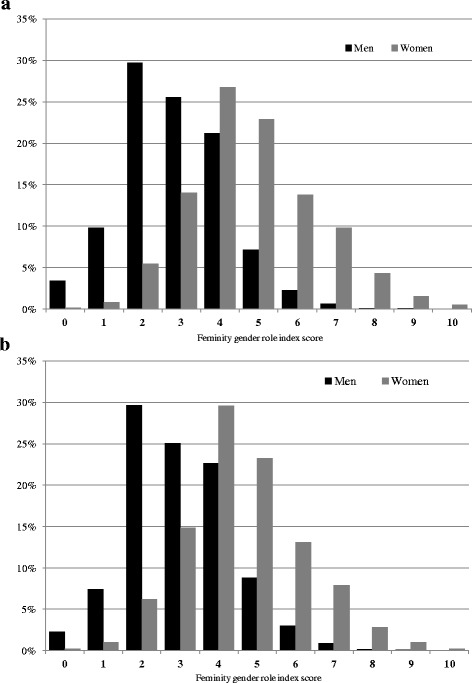
**a** Distribution of gender index score (higher scores = greater feminine gender roles) for Canadian men and women. 1997 Labour Force Survey. **b** Distribution of gender index score (higher scores = greater feminine gender roles) for Canadian men and women. 2014 Labour Force Survey

Figures 1a and b present the distribution of LFGI scores for men and women in 1997 (Fig. 1a) and 2014 (Fig. 1b). The distribution of LFGI scores was relatively similar for men and women in 1997 and 2014, with women scoring higher (more feminine) on the LFGI than men. It is important to note, in each year males and females were represented across the range of LFGI scores from 0 to 10, highlighting the distinction between gender as measured by the LFGI and biological sex.

Table 2 presents the polychoric correlations between the LFGI and its four component measures. Correlations for respondents in 1997 are presented below the diagonal and correlations for respondents in 2014 are presented above the diagonal. The relationship between the LFGI and its component measures were similar at both time points. The LFGI was most strongly correlated with occupation segregation and hours of work, and weakly correlated with education. Focusing on the measures included in the LFGI the highest correlation was observed between caring for children and hours of work in each survey year. Correlations indicated no redundancy between measures.

**Table 2:** Polychoric correlations between gender index and its components

	1	2	3	4	5
1. Gender Index	1.00	0.64	0.79	0.75	0.35
2. Responsibility for caring for children	0.72	1.00	0.21	0.35	-0.05
3. Occupation (based on 1997 LFS only)	0.79	0.27	1.00	0.20	-0.08
4. Hours of work	0.78	0.46	0.26	1.00	0.00
5. Education	0.43	0.03	-0.01	0.06	1.00

Correlations below diagonal are for 1997 LFS. Correlations above diagonal are for 2014 LFS

Table 3 presents the results of the linear regression model examining the interaction between sex and survey year on LFGI index scores after adjustment for age, province of residence and survey month. A statistically significant interaction was observed between sex and survey year. Although women had higher gender scores than men and gender scores increased between 1997 and 2014, this increase was not the same for men and women. To examine this interaction further, separate models were constructed for men and women. These models demonstrated that LFGI scores increased (indicating higher feminine gender roles) for men between 1997 and 2014, but decreased for women during the same time period (results not shown but available on request). The adjusted mean scores for the gender index for men increased from 2.81 in 1997 to 3.01 in 2014. For women the adjusted mean scores for the gender index decreased from 4.78 in 1997 to 4.64 in 2014.

**Table 3:** Adjusted ordinary least squared (OLS) estimates for sex, survey year and their interaction in gender index score

	Est	se	*p*-value
Sex			
Male	ref		
Female	1.57	0.01	< 0.001
Survey Year			
2014	ref		
1997	-0.21	0.01	< 0.001
Interaction			
Survey year/sex multiplicative interaction term	0.37	0.02	< 0.001

Respondents to the 1997 and 2014 LFS (*N* = 142,558; 10 % random sample)

*Est* OLS regression estimate, *se* standard error

Estimates additionally adjusted for age, age2, province/territory of residence and survey month

## Discussion

Disentangling the impacts of sex and gender in understanding male and female differences is increasingly recognised as an important aspect for advancing research and addressing knowledge gaps in the field of work-health [5]. However, achieving this goal in secondary data analyses where direct measures of gender, such as the BRSI or PAQ, have not been collected is challenging. The objective of this paper was to demonstrate how a gender index – based primarily on gender roles – could be developed using routinely collected information from the Canadian Labour Force Survey. A second objective was to examine how gender scores were distributed among men and women (i.e. sex) and if there had been changes in gender roles among working Canadian men and women over the 17 year period between 1997 and 2014. Differences were observed between men and women in each component of the LFGI, with women generally having higher LFGI scores (indicating greater feminine gender roles) compared to men. While we found that women had higher LFGI scores in both 1997 and 2014 small increases in LFGI scores were observed for men between 1997 and 2014, and small decreases in LFGI scores for women over the same time period.

These study results should be interpreted taking the following strengths and limitations into account. The household-based sampling strategy employed by Statistics Canada in conducting the LFS resulted in a truly representative sample of the Canadian labour market, and findings can be generalised to Canadian labour market participants over the study time period. However, the large sample also increases the possibility of a Type I error and the inference of a meaningful difference that has no practical or meaningful importance. This may be the case for the observed differences in the LFGI score over time and the interpretation that men are taking on greater feminine gender roles while women are taking on greater masculine gender roles. In each of these cases the differences over time periods were less than 0.5 on an index that ranges from 0 to 10. To put this into context, if LFGI scores continue to increase among men and decrease among women at the same rate as observed over the 19-year study period (1997 to 2014), it will take until 2097 for men and women to have similar LFGI scores, indicating gender-equity in relation to labour market roles.

Three of the four measures that comprised the LFGI distinguished between social and occupational roles of men and women in the expected direction. However, a similar number of men and women had lower education than their partner/spouse in 1997, while men were more likely than women to have lower education than their partner/spouse in 2014. To some extent this result reflects the changing nature of characteristics previously thought of as masculine or feminine [24]. For example, the BRSI has “ambitious” and “analytical” as masculine traits [9] while the PAQ has “likes math and science” and “intellectual” as masculine traits [12]. Given changes in educational participation between men and women, along with the information presented in this paper, future work that creates indexes/measures that reflect gender roles and institutionalised gender may choose to exclude education (as both an absolute measure and in relation to the respondent’s partner/spouse) as a component of such measures.

Finally, the construction of the LFGI represents the sum of scores for the relative components. While this approach has the advantage of simplicity, making the approach easy to replicate, it does make assumptions about the relative contribution of each of the components of the index in relation to overall labour market gender roles, which may not be valid. Alternative approaches to constructing the index (e.g. factor analyses or cluster analyses) may be warranted, and researchers should weigh the advantages and disadvantages to each analytic approach if they choose to replicate the work in this paper.

## How could the LFGI be used to better understand processes that create male/female differences in health?

**Fig. 2:**
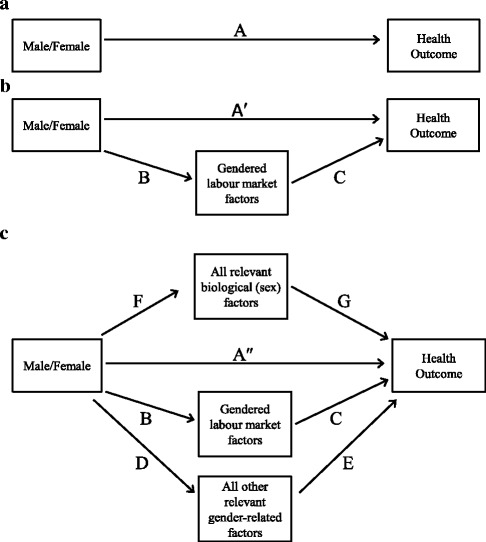
**a** A simple DAG linking male/female to a health outcome of interest. **b** An extended DAG to include gendered labour market factors (as measured by the LFGI). **c** A complete DAG to examine the factors that contribute to male/female differences in a given health outcome

While the LFS provided us with the most representative annual estimates for the Canadian labour market, it does not contain information on health indicators. This information, if available could have been used to further demonstrate how the LFGI might be applied to research to examine male/female differences in health status. To address this gap, a conceptual overview of how the LFGI might be included and interpreted in analyses using secondary data is provided below (Fig. 2a-c). We do this using directed acyclic graphs (DAGs), which provide a useful approach to understanding the causal relationships between variables and interpretation of effects in epidemiological analyses [25–27].

Figure 2a presents a simple DAG where there is a difference in a health outcome for men compared to women (note this difference could be in either direction – i.e. more prevalent among men compared to women, or more prevalent among women compared to men). If a difference in the health outcome is present among men and women, path “A” in this DAG will be not equal to zero and will be statistically significant. For theoretical purposes, the relationship between male/female and the health outcome (path A) is assumed to be adjusted for all confounders, and that male/female and the health outcome (along with the additional variables included in Fig. 2b and c below) have been measured without error.

In order to understand why the risk of the health outcome is greater for men (or women), the DAG is extended to include intermediate or mediating variables, such as the LFGI, to understand the impact of “sex” and “gender” in male/female differences in the health outcome. This extended model is presented in Fig. 2b. Again, for theoretical purposes, we make no confounding for all paths and no measurement error assumptions for all variables. We now have a direct path (path A′) and an indirect path (paths B and C) linking male/female to the health outcome. The magnitude of the indirect path will be determined by the strength of the relationship between male/female at the LFGI (path B) and the relationship between the LFGI and the health outcome (path C). The estimate for path B is equivalent to the regression estimate for female (relative to male) presented in Table 3 previously. It is important to note that the estimate from Table 3 indicates that the LFGI score was strongly influenced, but not completely explained by whether the respondent was male or female. The estimate for the direct effect (path A′) can be interpreted as the difference in the health outcome for men compared to women that would remain if men and women had similar roles in relation to labour market status (i.e. if men and women had similar scores on the LFGI). The difference between path A and path A′ (which in an ordinary least-squared model will be equivalent for the product term of path B and C) [28], can be interpreted as the amount of the originally observed difference in the health outcome for men and women that can be explained by differences in labour market roles (as assessed by the LFGI) between men and women [27].

It is important to note that if path A′ is still associated with the health outcome then this indicates that male/female differences in the health outcome are not completely explained by differences in labour market roles only. The remaining differences between men and women (path A′) will likely be a combination of other biological (sex) differences between men and women that are relevant to the outcome, and other social (gender) differences between men and women that are both relevant to the outcome, and not captured in the LFGI. This has been explicitly described in Fig. 2c. If data was available to construct – either individually or as part of an index – all other sex and gender related factors that are relevant to the outcome of interest, then path A″ in Fig. 2c would approach zero, and one could examine the relative contribution of biological factors (paths F and G) and gender factors related to labour market roles (paths B and C) and non-labour market roles (paths D and E). The caveat for Fig. 2c is that each of the three pathways can be measured and estimated as distinct from each other. This highlights the need for the integration of “sex” and “gender” into the study design and data collection phase as part of a comprehensive research process [4], so that a more thorough examination of sex and gender into male/female differences in health status can be routinely undertaken using the approach outlined above.

## Conclusions

In this paper we developed a measure of feminine and masculine gender roles, using self-reported survey data, where no direct measure of gender (masculinity/femininity) was available. This measure had face validity in terms of being related to, but distinct from sex (male/female), and was also responsive to change over time. Future research should examine the relative importance of including additional measures to an index such as the LFGI. For example, in the study by Pelletier and colleagues [14] primary earner status was the measure most strongly related to masculine BRSI scores, while the number of hours spent doing housework and responsibility for doing housework were the measures most strongly associated with high feminine BRSI scores. The LFGI in this paper included some indication of primary earner status and some indication of household responsibilities for respondents. However, a more detailed or direct measure of primary earner status and household responsibilities (in particular housework) may have allowed further refinement or distinction between masculine and feminine roles in the LFGI. In addition, it would be interesting to examine how an index with a reduced number of measures would perform in differentiating gender roles for men and women. As mentioned in the introduction to this paper, the early work by Lippa and colleagues focused only on differences in occupational preferences between men and women [11, 13]. Interestingly, occupational segregation, as well as hours worked, was the measure most strongly correlated with the LFGI in our study. We also suggest that research examining work-related health outcomes should (and in many cases can) integrate measures of sex and gender, using an approach similar to the one outlined in this paper.

## Abbreviations

BSRI, Bem-Sex-Role-Inventory; LFGI, Labour Force Gender Index; LFS, Canadian Labour Force Survey; PAQ, personal attributes questionnaire.
